# Asymptomatic and Biochemically Silent Pheochromocytoma with Characteristic Findings on Imaging

**DOI:** 10.1155/2020/8847261

**Published:** 2020-09-26

**Authors:** Andrew Spiro, Aqueel Usman, Asif Ajmal, Thanh D. Hoang, Mohamed K. M. Shakir

**Affiliations:** Division of Endocrinology, Walter Reed National Military Medical Center, 8901 Wisconsin Ave., Bethesda, MD 20889, USA

## Abstract

Pheochromocytomas are tumors that originate from the chromaffin tissue of the adrenal medulla and commonly produce catecholamines. The diagnosis is typically established by the measurement of catecholamines or their metabolites in urine or plasma, and tumors are localized with the use of radiographic and scintigraphic studies. Pheochromocytomas can occur in asymptomatic patients, and the preferred treatment is surgical removal of the tumor. We report a 48-year-old male with a left adrenal incidentaloma, which progressively increased in size from 1.1 cm to 2.6 cm over a 4-year period, as measured by an adrenal computed tomography (CT) scan. Throughout his entire course of treatment, he was asymptomatic with normal blood pressure readings. His biochemical screening was unremarkable for the first three years of tumor surveillance. Follow-up imaging, including CT and MRI, showed findings suspicious for pheochromocytoma, and the diagnosis was ultimately made with the combination of imaging and laboratory studies. He underwent laparoscopic resection of the adrenal mass with confirmation of pheochromocytoma on histology. This case illustrates how CT and MRI findings can alert providers to the presence of a pheochromocytoma, even in an asymptomatic, biochemically negative patient.

## 1. Introduction

Catecholamine-producing tumors arise from chromaffin cells in the sympathoadrenal system. Approximately 80 to 85% of catecholamine-producing tumors originate from the adrenal medulla, and these are called pheochromocytomas. About 15 to 20% derive from the sympathetic paravertebral ganglia and are known as catecholamine-secreting paragangliomas. It is worth noting, that non-catecholamine-secreting paragangliomas usually arise from the parasympathetic ganglia in the neck and base of the skull. Among patients with hypertension in the outpatient setting, the prevalence of catecholamine-producing tumors is estimated to be 0.2% to 0.6% [1]. The adrenal medulla normally secretes about 80% epinephrine, but the tumors of the adrenal medulla predominantly secrete more norepinephrine than epinephrine [2]. The elevated catecholamines can give rise to symptoms including sustained or paroxysmal hypertension, headaches, palpitations, tremors, diaphoresis, dyspnea, anxiety, chest pain, nausea, vomiting, and paresthesia. Catecholamines also have anti-insulinemic actions, which can cause elevated blood glucose levels and may result it polydipsia and polyuria. In severe cases, patients may experience myocardial infarction, heart failure, pulmonary edema, arrhythmias, or intracranial hemorrhage. A diagnosis is usually established by measuring the levels of metanephrines in the urine or blood [3]. Computed tomography (CT) or magnetic resonance imaging (MRI) are used for tumor localization. The tumor can also present as an asymptomatic adrenal incidentaloma, identified radiographically. Here, we report one such interesting case.

## 2. Case Presentation

A 48-year-old male with a left adrenal incidentaloma was evaluated in the endocrine clinic in 2013. CT showed a 1.1 cm left adrenal mass, with attenuation of 55 Hounsfield Units (HU). He was completely asymptomatic, with blood pressure readings in the normal range. A biochemical evaluation was unremarkable, including urine and plasma metanephrine levels, 1 mg dexamethasone suppression test, and plasma aldosterone-to-renin ratio. Repeat biochemical evaluations in 2014 and 2015 were also unremarkable; however, the size of the adrenal mass increased to 1.5 cm in 2014 and to 1.8 cm in 2015.

**Figure 1:**
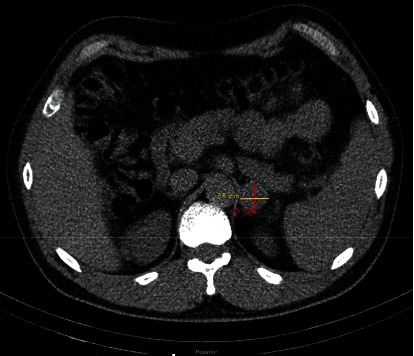
CT scan showing a 2.6 cm left adrenal mass.

**Figure 2:**
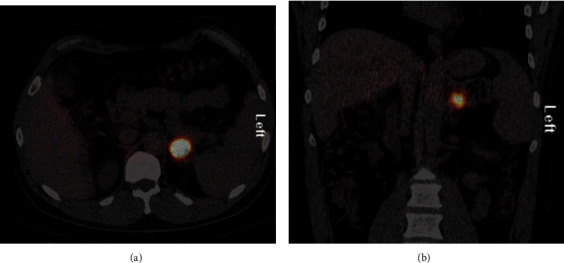
^123^I-metaiodobenzylguanidine (MIBG) scintigraphy scan showed abnormal focalintense radiotracer accumulation in the left adrenal lesion

**Figure 3:**
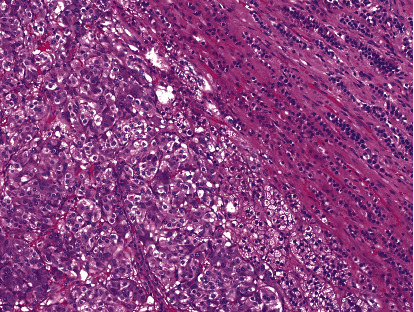
Histology demonstrates the well-circumscribed nature of the typical pheochromocytoma. There is an abrupt and distinct border between the pheochromocytoma on the left and the surrounding normal adrenal gland on the right. The tumor has the typical low-power architecture with cells arranged in nests and cords.

In 2016, adrenal CT (Figure 1) showed the mass had increased to 2.6 cm, with an attenuation of 40 HU. An MRI scan confirmed a 2.4 × 2.4 × 2.5 cm left adrenal mass with hyperintense T2 signal, isotense T1 signal without drop of signal on out-of-phase images, and homogenous enhancement postcontrast. The patient denied any symptoms consistent with pheochromocytoma. Physical examination was normal, including heart rate and blood pressure measurement. Plasma metanephrines and chromogranin A were unremarkable. A 24-hour urine metanephrine level was normal at 160 mcg (ref 45–290), while a 24-hour urine normetanephrine level was elevated at 1415 mcg (ref 82–500). An ^123^I-metaiodobenzylguanidine (MIBG) scintigraphy scan revealed abnormal focal intense radiotracer accumulation in the left adrenal lesion (Figures 2(a) and 2(b)). With the presumptive diagnosis of pheochromocytoma, the patient was treated with phenoxybenzamine and underwent laparoscopic resection of the left adrenal mass. A 3.0 × 2.6 × 2.5 cm left adrenal pheochromocytoma was confirmed on histology (Figure 3). Postoperatively, the patient continued to be asymptomatic and normotensive, with normal levels of serum and urine metanephrine levels.

## 3. Discussion

Our patient demonstrated an asymptomatic incidentaloma that was biochemically negative for the first three years after it was identified. The diagnosis of pheochromocytoma was ultimately made with the combination of follow-up imaging and lab work. In addition to curing hypertension, the early diagnosis of pheochromocytoma is important because if unrecognized, it can be a lethal condition. Medications, anesthetic agents, pregnancy, and surgery can precipitate hypertensive crisis or shock, even in asymptomatic patients [4]. The widespread use of radiological imaging has increased the detection of adrenal incidentalomas in individuals over 30 years of age. The clinical parameters of the tumor that need to be considered include functionality and aggressiveness. Assessing functionality involves identifying the secretion of cortisol, aldosterone, catecholamines, and androgens [5]. A retrospective study evaluated 42 patients with adrenal incidentalomas who were treated between 1995 and 2005, to determine the diagnostic significance of borderline-elevated urine or plasma metanephrines, defined as 1-2 times the upper limit of normal. The study found that 30% of the patients with adrenal incidentalomas and borderline-elevated urine or plasma metanephrines had a pheochromocytoma [6]. In another study of 27 patients with pheochromocytomas, 19 patients (70%) had an incidentally discovered tumor, six patients (22%) were normotensive, and two patients had near-normal urinary metanephrine levels [7]. For three years after the adrenal incidentaloma was identified, our patient's biochemical parameters, including urine and plasma metanephrine levels, were normal. Four years after the identification of the incidentaloma, it had significantly increased in size, and the 24-hour urine normetanephrine level was elevated; however, 24-hour urine metanephrine values remained normal, and serum metanephrine levels were never elevated.

In general, a CT scan with contrast-enhancement and MRI are used to localize pheochromocytomas [8]. The Endocrine Society Guidelines recommend CT rather than MRI as the initial imaging modality for most patients, due to excellent spacial resolution in the thorax, abdomen, and pelvis [1]. A retrospective study of 41 patients, who had undergone surgery for pheochromocytoma between 1990 and 2002, found that the classic association of headaches, palpitations, and sweating was only present in 24% of the cases. In roughly half of the cases, the tumor was discovered incidentally by imaging that was not part of a diagnostic workup for abnormal blood pressure readings [9]. Several imaging findings can be indicative of pheochromocytoma, adrenocortical carcinoma, or metastasis, including heterogeneity, cystic or hemorrhagic changes, high Hounsfield density (>10 HU) on CT, marked enhancement with intravenous contrast, delayed contrast washout (less than 60% at 10 minutes), or a high signal intensity on a T2-weighted MRI. It should be noted that a pheochromocytoma with lipid degeneration can result in low attenuation scores (<10 HU) and more than 60% washout in delayed CT scanning, which can appear similar to adrenal adenomas [10]. Characteristic features of benign adrenal adenomas include a size less than 5 cm, sharp margins, lack of significant growth on serial imaging, low attenuation scores, and more than 60% washout by delayed CT [8, 11]. Our patient's initial imaging was not concerning for pheochromocytoma, largely because of the small size of the mass. Serial CT imaging showed a progressive increase in size, and the most recent scan showed a Hounsfield density of 40 HU. Additionally, MRI showed a hyperintense T2 signal, isotense T1 signal without drop of signal on out-of-phase images, and homogenous enhancement postcontrast.

In our patient, ^123^I-MIBG scintigraphy showed abnormal focal radiotracer accumulation in the left adrenal lesion with no other foci. MIBG is a compound that resembles norepinephrine and is actively concentrated within the sympathomedullary tissue. ^123^I-MIBG scintigraphy may be useful for identification of tumors missed by MRI or CT. It can also be used for identifying catecholamine-secreting paragangliomas, multifocality, or multiple smaller masses throughout the body, such as metastasis [12, 13].

One contributing factor for our patient's initial negative biochemical profile could be the relatively small tumor size. The average size of pheochromocytomas at the time of diagnosis is 4.9 cm, and there is evidence that tumor size correlates with the hormone level [14]. Even with relatively large pheochromocytomas though, there are reasons why a patient may be asymptomatic. A large tumor can have only a small amount of functional tissue, or rapid turnover in the tumor may result in the release of predominantly metabolized catecholamines. A tumor may be stress-activated or only secrete catecholamines episodically. There can also be false-negative laboratory results because of handling the sample at a high room temperature or due to caffeine ingestion within 24 hours prior to testing [15].

## 4. Conclusions

The early diagnosis of pheochromocytoma is important because if unrecognized, it can lead to high morbidity and mortality. Our patient demonstrated an asymptomatic incidentaloma that was biochemically negative for the first few years after identification. Follow-up imaging, including CT and MRI, showed findings suspicious for pheochromocytoma, and the diagnosis was ultimately made with the combination of imaging and laboratory studies. This case shows how CT and MRI findings can alert providers to the presence of a pheochromocytoma, even in an asymptomatic, biochemically negative patient.

## Data Availability

No data were used to support this study.
